# 2-[(4-Meth­oxy­anilino)meth­yl]phenol

**DOI:** 10.1107/S1600536811031679

**Published:** 2011-08-11

**Authors:** Hong Shu, Ning-Shu Yu, Guo-Lan Xie, Li-Zhuang Chen

**Affiliations:** a School of Biology and Chemical Engineering, Jiangsu University of Science and Technology, Zhenjiang 212003, People’s Republic of China

## Abstract

In the title compound, C_14_H_15_NO_2_, the dihedral angle between the two benzene rings is 71.10 (5)°. In the crystal, mol­ecules are linked by inter­molecular N—H⋯O, and O—H⋯N hydrogen bonds into a chain running parallel to the *b* axis.

## Related literature

For the synthesis of the title compound, see: Noda (1959 ▶). For other related structures, see: Liu *et al.* (2007 ▶); Qu *et al.* (2007 ▶).
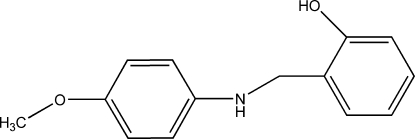


## Experimental

### 

#### Crystal data

C_14_H_15_NO_2_
*M* *_r_* = 229.27
Monoclinic, 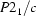

*a* = 7.8132 (16) Å
*b* = 5.7947 (12) Å
*c* = 26.175 (5) Å
β = 95.02 (3)°
*V* = 1180.5 (4) Å^3^
*Z* = 4
Mo *K*α radiation
μ = 0.09 mm^−1^
*T* = 298 K
0.40 × 0.30 × 0.20 mm

#### Data collection

Rigaku SCXmini diffractometer
Absorption correction: multi-scan (*CrystalClear*; Rigaku, 2005 ▶) *T*_min_ = 0.970, *T*_max_ = 0.983
10971 measured reflections
2693 independent reflections
1692 reflections with *I* > 2σ(*I*)
*R*_int_ = 0.056

#### Refinement

*R*[*F* ^2^ > 2σ(*F* ^2^)] = 0.056
*wR*(*F* ^2^) = 0.128
*S* = 1.03
2693 reflections
163 parameters
2 restraints
H atoms treated by a mixture of independent and constrained refinement
Δρ_max_ = 0.17 e Å^−3^
Δρ_min_ = −0.22 e Å^−3^

### 

Data collection: *CrystalClear* (Rigaku, 2005 ▶); cell refinement: *CrystalClear*; data reduction: *CrystalClear*; program(s) used to solve structure: *SHELXS97* (Sheldrick, 2008 ▶); program(s) used to refine structure: *SHELXL97* (Sheldrick, 2008 ▶); molecular graphics: *SHELXTL* (Sheldrick, 2008 ▶); software used to prepare material for publication: *SHELXL97*.

## Supplementary Material

Crystal structure: contains datablock(s) I, global. DOI: 10.1107/S1600536811031679/pv2441sup1.cif

Structure factors: contains datablock(s) I. DOI: 10.1107/S1600536811031679/pv2441Isup2.hkl

Supplementary material file. DOI: 10.1107/S1600536811031679/pv2441Isup3.cml

Additional supplementary materials:  crystallographic information; 3D view; checkCIF report

## Figures and Tables

**Table 1:** Hydrogen-bond geometry (Å, °)

*D*—H⋯*A*	*D*—H	H⋯*A*	*D*⋯*A*	*D*—H⋯*A*
N1—H1⋯O2^i^	0.86 (1)	2.22 (1)	3.058 (2)	163 (2)
O2—H2⋯N1^ii^	0.86 (1)	1.89 (1)	2.741 (2)	172 (2)

Symmetry codes: (i) 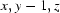; (ii) 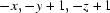.

**Fig. 1.**
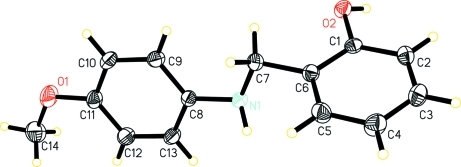
The asymmetric unit of the title compound with atom labels. Displacement ellipsoids are drawn at the 30% probability level.

**Fig. 2.**
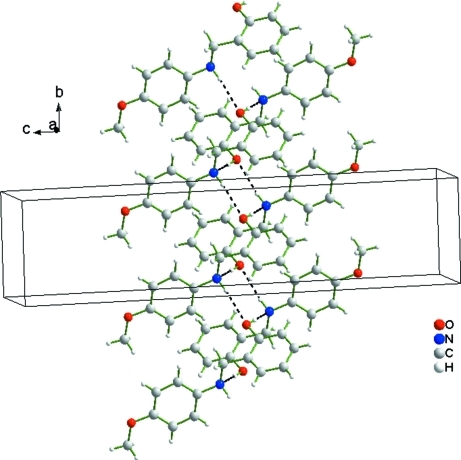
The unit cell packing of the title compound viewed along the b-axis. Hydrogen bonds are drawn as dashed lines.

## supplementary crystallographic information

### Comment

Recently, the crystal structures of compounds closely related to the title molecule, *e.g*., 2-[(4-chlorophenyl)aminomethyl]-6-methoxyphenol (Liu *et al.*, 2007) and 2-(anilinomethyl)phenol (Qu *et al.*, 2007) have been reported. We report here the crystal structure of a new member of this family of compounds.

In the title compound (Fig. 1), the dihedral angle between the two benzene ring planes is 71.10 (5)°. In the crystal structure, the molecules are linked by intermolecular N—H···O, and O—H···N hydrogen bonds into a one-dimensional chain lying parallel to the *b*-axis (Fig. 2).

### Experimental

The title compound was synthesized by the reaction of 2-((4-methoxyphenylimino)- methyl)phenol (2.76 g, 10 mmol) with NaBH_4_ (0.38 g, 10 mmol) in methanol (50 ml) according to the reported method (Noda, 1959). Crystals were obtained from an ethanolic (95%) solution by slow evaporation at room temperature.

### Refinement

H atoms were placed at calculated positions and were included in the refinement in the riding-model approximation, with C—H = 0.93, 0.96 and 0.97 Å, for aryl, methyl and methylene H-atoms, respectively, with *U*_iso_(H) = 1.2 (or 1.5 for methyl) *U*_eq_(C). The hydrogen atoms bonded to O and N were included in the positions obtained from a difference map and were allowed to refine with distances contarined at N—H and O—H = 0.86 (1) Å.

### Crystal data

**Table:**

C_14_H_15_NO_2_	*F*(000) = 488
*M**_r_* = 229.27	*D*_x_ = 1.290 Mg m^−^^3^
Monoclinic, *P*2_1_/*c*	Mo *K*α radiation, λ = 0.71073 Å
Hall symbol: -P 2ybc	Cell parameters from 8657 reflections
*a* = 7.8132 (16) Å	θ = 3.1–27.5°
*b* = 5.7947 (12) Å	µ = 0.09 mm^−^^1^
*c* = 26.175 (5) Å	*T* = 298 K
β = 95.02 (3)°	Prism, colorless
*V* = 1180.5 (4) Å^3^	0.40 × 0.30 × 0.20 mm
*Z* = 4	

### Data collection

**Table:**

Rigaku SCXmini diffractometer	2693 independent reflections
Radiation source: fine-focus sealed tube	1692 reflections with *I* > 2σ(*I*)
graphite	*R*_int_ = 0.056
Detector resolution: 13.6612 pixels mm^-1^	θ_max_ = 27.5°, θ_min_ = 3.1°
CCD_Profile_fitting scans	*h* = −9→10
Absorption correction: multi-scan (*CrystalClear*; Rigaku, 2005)	*k* = −7→7
*T*_min_ = 0.970, *T*_max_ = 0.983	*l* = −33→33
10971 measured reflections	

### Refinement

**Table:**

Refinement on *F*^2^	Primary atom site location: structure-invariant direct methods
Least-squares matrix: full	Secondary atom site location: difference Fourier map
*R*[*F*^2^ > 2σ(*F*^2^)] = 0.056	Hydrogen site location: inferred from neighbouring sites
*wR*(*F*^2^) = 0.128	H atoms treated by a mixture of independent and constrained refinement
*S* = 1.03	*w* = 1/[σ^2^(*F*_o_^2^) + (0.0527*P*)^2^ + 0.1931*P*] where *P* = (*F*_o_^2^ + 2*F*_c_^2^)/3
2693 reflections	(Δ/σ)_max_ < 0.001
163 parameters	Δρ_max_ = 0.17 e Å^−^^3^
2 restraints	Δρ_min_ = −0.22 e Å^−^^3^

### Special details

**Table:**

Geometry. All e.s.d.'s (except the e.s.d. in the dihedral angle between two l.s. planes) are estimated using the full covariance matrix. The cell e.s.d.'s are taken into account individually in the estimation of e.s.d.'s in distances, angles and torsion angles; correlations between e.s.d.'s in cell parameters are only used when they are defined by crystal symmetry. An approximate (isotropic) treatment of cell e.s.d.'s is used for estimating e.s.d.'s involving l.s. planes.
Refinement. Refinement of *F*^2^ against ALL reflections. The weighted *R*-factor *wR* and goodness of fit *S* are based on *F*^2^, conventional *R*-factors *R* are based on *F*, with *F* set to zero for negative *F*^2^. The threshold expression of *F*^2^ > σ(*F*^2^) is used only for calculating *R*-factors(gt) *etc*. and is not relevant to the choice of reflections for refinement. *R*-factors based on *F*^2^ are statistically about twice as large as those based on *F*, and *R*- factors based on ALL data will be even larger.

### Fractional atomic coordinates and isotropic or equivalent isotropic displacement parameters (Å^2^)

**Table:**

	*x*	*y*	*z*	*U*_iso_*/*U*_eq_	
O2	0.12511 (17)	0.7060 (2)	0.48819 (5)	0.0369 (4)	
N1	0.1955 (2)	0.1501 (3)	0.54954 (6)	0.0322 (4)	
C8	0.2161 (2)	0.0685 (3)	0.60126 (6)	0.0301 (4)	
C7	0.3137 (2)	0.3330 (3)	0.53561 (7)	0.0350 (5)	
H7A	0.4309	0.2888	0.5467	0.042*	
H7B	0.2883	0.4746	0.5532	0.042*	
C11	0.2449 (3)	−0.1048 (4)	0.70119 (7)	0.0395 (5)	
C6	0.2976 (2)	0.3745 (3)	0.47851 (6)	0.0317 (4)	
C1	0.2033 (2)	0.5582 (3)	0.45649 (6)	0.0295 (4)	
C9	0.3148 (2)	0.1824 (3)	0.64015 (7)	0.0370 (5)	
H9	0.3717	0.3180	0.6330	0.044*	
O1	0.2685 (2)	−0.1765 (3)	0.75158 (5)	0.0600 (5)	
C2	0.1942 (2)	0.5952 (3)	0.40425 (7)	0.0365 (5)	
H2A	0.1322	0.7199	0.3900	0.044*	
C10	0.3290 (3)	0.0950 (3)	0.68955 (7)	0.0410 (5)	
H10	0.3961	0.1721	0.7152	0.049*	
C13	0.1307 (3)	−0.1295 (3)	0.61362 (7)	0.0397 (5)	
H13	0.0618	−0.2056	0.5882	0.048*	
C5	0.3797 (3)	0.2304 (3)	0.44614 (8)	0.0442 (5)	
H5	0.4435	0.1067	0.4601	0.053*	
C12	0.1450 (3)	−0.2177 (4)	0.66294 (7)	0.0430 (5)	
H12	0.0874	−0.3525	0.6703	0.052*	
C4	0.3699 (3)	0.2647 (4)	0.39393 (8)	0.0488 (6)	
H4	0.4257	0.1649	0.3731	0.059*	
C3	0.2769 (3)	0.4478 (4)	0.37300 (7)	0.0437 (5)	
H3	0.2696	0.4726	0.3378	0.052*	
C14	0.1791 (4)	−0.3750 (4)	0.76569 (8)	0.0698 (8)	
H14A	0.0576	−0.3478	0.7603	0.105*	
H14B	0.2096	−0.4089	0.8012	0.105*	
H14C	0.2092	−0.5034	0.7451	0.105*	
H1	0.195 (2)	0.032 (2)	0.5294 (6)	0.046 (6)*	
H2	0.0288 (19)	0.756 (4)	0.4738 (9)	0.083 (9)*	

### Atomic displacement parameters (Å^2^)

**Table:**

	*U*^11^	*U*^22^	*U*^33^	*U*^12^	*U*^13^	*U*^23^
O2	0.0365 (8)	0.0393 (8)	0.0342 (7)	0.0069 (7)	−0.0009 (6)	−0.0054 (6)
N1	0.0345 (9)	0.0315 (9)	0.0301 (9)	−0.0004 (7)	−0.0006 (7)	−0.0026 (7)
C8	0.0285 (10)	0.0322 (10)	0.0295 (9)	0.0057 (8)	0.0021 (8)	−0.0013 (8)
C7	0.0339 (11)	0.0342 (11)	0.0358 (10)	0.0013 (9)	−0.0029 (8)	0.0008 (8)
C11	0.0444 (12)	0.0466 (12)	0.0278 (10)	0.0049 (10)	0.0054 (9)	0.0003 (9)
C6	0.0299 (10)	0.0330 (10)	0.0319 (10)	−0.0002 (8)	0.0016 (8)	0.0000 (8)
C1	0.0267 (9)	0.0296 (10)	0.0325 (10)	−0.0044 (8)	0.0037 (8)	−0.0040 (8)
C9	0.0397 (11)	0.0355 (11)	0.0360 (11)	−0.0041 (9)	0.0036 (9)	−0.0029 (8)
O1	0.0814 (12)	0.0676 (11)	0.0304 (8)	−0.0100 (9)	0.0011 (8)	0.0072 (7)
C2	0.0355 (11)	0.0379 (11)	0.0355 (10)	0.0016 (9)	0.0005 (9)	0.0046 (9)
C10	0.0441 (12)	0.0468 (12)	0.0309 (10)	−0.0020 (10)	−0.0029 (9)	−0.0083 (9)
C13	0.0436 (12)	0.0420 (12)	0.0326 (10)	−0.0072 (9)	−0.0015 (9)	−0.0043 (9)
C5	0.0486 (13)	0.0406 (12)	0.0434 (12)	0.0136 (10)	0.0038 (10)	0.0013 (9)
C12	0.0496 (13)	0.0424 (12)	0.0372 (11)	−0.0101 (10)	0.0057 (10)	0.0023 (9)
C4	0.0520 (13)	0.0541 (14)	0.0415 (12)	0.0128 (11)	0.0106 (10)	−0.0083 (10)
C3	0.0455 (12)	0.0547 (13)	0.0317 (10)	−0.0011 (11)	0.0073 (9)	0.0000 (9)
C14	0.111 (2)	0.0580 (16)	0.0417 (13)	−0.0056 (15)	0.0122 (14)	0.0121 (11)

### Geometric parameters (Å, °)

**Table:**

O2—C1	1.372 (2)	C9—H9	0.9300
O2—H2	0.862 (10)	O1—C14	1.412 (3)
N1—C8	1.430 (2)	C2—C3	1.382 (3)
N1—C7	1.472 (2)	C2—H2A	0.9300
N1—H1	0.864 (9)	C10—H10	0.9300
C8—C13	1.380 (3)	C13—C12	1.384 (3)
C8—C9	1.389 (2)	C13—H13	0.9300
C7—C6	1.508 (2)	C5—C4	1.376 (3)
C7—H7A	0.9700	C5—H5	0.9300
C7—H7B	0.9700	C12—H12	0.9300
C11—C10	1.378 (3)	C4—C3	1.372 (3)
C11—C12	1.379 (3)	C4—H4	0.9300
C11—O1	1.380 (2)	C3—H3	0.9300
C6—C5	1.386 (3)	C14—H14A	0.9600
C6—C1	1.390 (2)	C14—H14B	0.9600
C1—C2	1.380 (2)	C14—H14C	0.9600
C9—C10	1.384 (3)		
			
C1—O2—H2	111.4 (17)	C1—C2—H2A	119.8
C8—N1—C7	116.84 (14)	C3—C2—H2A	119.8
C8—N1—H1	108.1 (13)	C11—C10—C9	120.88 (18)
C7—N1—H1	112.8 (13)	C11—C10—H10	119.6
C13—C8—C9	118.17 (17)	C9—C10—H10	119.6
C13—C8—N1	118.71 (16)	C8—C13—C12	121.63 (18)
C9—C8—N1	123.10 (17)	C8—C13—H13	119.2
N1—C7—C6	111.14 (15)	C12—C13—H13	119.2
N1—C7—H7A	109.4	C4—C5—C6	122.09 (19)
C6—C7—H7A	109.4	C4—C5—H5	119.0
N1—C7—H7B	109.4	C6—C5—H5	119.0
C6—C7—H7B	109.4	C11—C12—C13	119.77 (19)
H7A—C7—H7B	108.0	C11—C12—H12	120.1
C10—C11—C12	119.22 (18)	C13—C12—H12	120.1
C10—C11—O1	115.96 (18)	C3—C4—C5	119.31 (19)
C12—C11—O1	124.81 (19)	C3—C4—H4	120.3
C5—C6—C1	117.69 (17)	C5—C4—H4	120.3
C5—C6—C7	120.43 (17)	C4—C3—C2	120.01 (18)
C1—C6—C7	121.88 (16)	C4—C3—H3	120.0
O2—C1—C2	121.01 (16)	C2—C3—H3	120.0
O2—C1—C6	118.35 (15)	O1—C14—H14A	109.5
C2—C1—C6	120.59 (16)	O1—C14—H14B	109.5
C10—C9—C8	120.31 (18)	H14A—C14—H14B	109.5
C10—C9—H9	119.8	O1—C14—H14C	109.5
C8—C9—H9	119.8	H14A—C14—H14C	109.5
C11—O1—C14	117.88 (17)	H14B—C14—H14C	109.5
C1—C2—C3	120.31 (18)		
			
C7—N1—C8—C13	−168.27 (16)	C6—C1—C2—C3	0.9 (3)
C7—N1—C8—C9	13.4 (2)	C12—C11—C10—C9	−0.3 (3)
C8—N1—C7—C6	170.60 (14)	O1—C11—C10—C9	179.82 (18)
N1—C7—C6—C5	−80.6 (2)	C8—C9—C10—C11	−0.5 (3)
N1—C7—C6—C1	100.3 (2)	C9—C8—C13—C12	−1.6 (3)
C5—C6—C1—O2	−178.27 (16)	N1—C8—C13—C12	−179.97 (17)
C7—C6—C1—O2	0.8 (3)	C1—C6—C5—C4	−0.1 (3)
C5—C6—C1—C2	−0.6 (3)	C7—C6—C5—C4	−179.12 (19)
C7—C6—C1—C2	178.44 (17)	C10—C11—C12—C13	0.1 (3)
C13—C8—C9—C10	1.3 (3)	O1—C11—C12—C13	179.99 (18)
N1—C8—C9—C10	179.69 (17)	C8—C13—C12—C11	0.8 (3)
C10—C11—O1—C14	177.40 (19)	C6—C5—C4—C3	0.4 (3)
C12—C11—O1—C14	−2.5 (3)	C5—C4—C3—C2	−0.1 (3)
O2—C1—C2—C3	178.53 (17)	C1—C2—C3—C4	−0.6 (3)

### Hydrogen-bond geometry (Å, °)

**Table:**

*D*—H···*A*	*D*—H	H···*A*	*D*···*A*	*D*—H···*A*
N1—H1···O2^i^	0.86 (1)	2.22 (1)	3.058 (2)	163 (2)
O2—H2···N1^ii^	0.86 (1)	1.89 (1)	2.741 (2)	172 (2)

Symmetry codes: (i) *x*, *y*−1, *z*; (ii) −*x*, −*y*+1, −*z*+1.

## References

[bb1] Liu, Y.-F., Xia, H.-T., Yang, S.-P. & Wang, D.-Q. (2007). *Acta Cryst.* E**63**, o3561.

[bb2] Noda, M. (1959). *J. Org. Chem.* **24**, 1209–1212.

[bb3] Qu, Y., Tian, L.-J. & Dong, J. (2007). *Acta Cryst.* E**63**, o4832.

[bb4] Rigaku (2005). *CrystalClear* Rigaku Corporation, Tokyo, Japan.

[bb5] Sheldrick, G. M. (2008). *Acta Cryst.* A**64**, 112–122. 10.1107/S0108767307043930 18156677

